# Growth and Hepatopancreas Health of Juvenile Chinese Mitten Crab (*Eriocheir sinensis*) Fed Different Levels of Black Soldier Fly (*Hermetia illucens*) Larvae Meal for Fish Meal Replacement

**DOI:** 10.1155/2024/6625061

**Published:** 2024-01-23

**Authors:** Han Wang, Erchao Li, Qincheng Huang, Jiadai Liu, Yixin Miao, Xiaodan Wang, Chuanjie Qin, Jianguang Qin, Liqiao Chen

**Affiliations:** ^1^Laboratory of Aquaculture Nutrition and Environmental Health, School of Life Sciences, East China Normal University, Shanghai 200241, China; ^2^Xianghu Laboratory, Hangzhou 311231, China; ^3^Key Laboratory of Sichuan Province for Fishes Conservation and Utilization in the Upper Reaches of the Yangtze River, Neijiang Normal University, Neijiang, Sichuan 641100, China; ^4^College of Science and Engineering, Flinders University, Adelaide, South Australia 5001, Australia

## Abstract

A 56-day feeding trial assessed the effects of black soldier fly larvae meal (BSFLM) on the growth performance and hepatopancreas health of juvenile *Eriocheir sinensis*. Six isoproteic and isolipidic diets with 0% (FM), 10% (BSFLM10), 20% (BSFLM20), 30% (BSFLM30), 40% (BSFLM40), or 50% (BSFLM50) replacement of fish meal by BSFLM were formulated. Compared to FM, replacing 10%–40% of fish meal with BSFLM did not significantly affect the weight gain rate (WGR) or specific growth rate (SGR), while BSFLM50 significantly decreased the WGR and SGR. Crabs fed BSFLM50 had significantly lower T-AOC activity than those fed other diets, and crabs fed BSFLM30, BSFLM40, or BSFLM50 had significantly lower activities of antioxidant enzymes (SOD and GSH-Px) in the hepatopancreas than those fed FM or BSFLM10. Compared to FM, BSFLM10, BSFLM20, and BSFLM30 did not affect the relative expression of genes related to the nonspecific immunity, while BSFLM40 and BSFLM50 upregulated the relative expression of these genes. Furthermore, histological analysis showed that the hepatopancreas was deformed in the BSFLM50 group, with widened lumens and loss of basal membrane integrity. In summary, BSFLM replacing 50% of fish meal reduced growth and structural damage to the hepatopancreas. An immune response was activated when the replacement level was over 30%. Therefore, the replacement level of dietary fish meal by BSFLM is recommended to be not more than 30% of the juvenile *E. sinensis* feed.

## 1. Introduction

Fish meal is considered the primary protein source of commercial aquatic feeds due to its high-protein content, balanced amino acid composition, high quality of lipids, and unknown growth promoters [1]. The decline in the output of fish meal and the increased demand for the aquaculture feeds have resulted in its increased concurrent price [2]. Therefore, developing alternative protein sources for fish meal has been a hot research topic in the aquaculture [3]. In recent years, insect protein has attracted significant attention due to its wide range of sources, easy accessibility, abundance of nutrients, and environmental friendliness [4–6]. The use of insect proteins in the aquatic animal feed has been widely reported. A study showed that fly (*Musca domestica*) maggot meal could replace 100% of fish meal without negatively affecting growth performance and nutrient utilization in African catfish (*Clarias gariepinus*) fingerlings [7]. The replacement of fish meal by defatted yellow mealworm (*Tenebrio molitor*) improved the growth and immunity of Pacific white shrimp (*Litopenaeus vannamei*) [8]. These studies demonstrated that insect protein has enormous potential to replace fish meal in the feeds of aquatic animals. More products of different insect species need to be evaluated before expanding the variety of insect proteins.

The black soldier fly (*Hermetia illucens*) is a fly (Diptera) of the Stratiomyidae family. It was initially reared to dispose of the organic wastes. Larvae can convert waste into protein-rich and fat-rich biomass, making the culture of black soldier flies environmentally friendly and inexpensive [9, 10]. Black soldier fly larvae meal (BSFLM) has a more similar amino acid pattern to fish meal than other insect protein resources and is expected to be an alternative to fish meal [11, 12]. A 56-day feeding trial on Jian carp (*Cyprinus carpio var. Jian*) showed that the growth, biological parameters, proximate composition, amino acid composition, and serum biochemical parameters were not affected by BSFLM replacement [13]. However, the lipid content of black soldier fly larvae is highly variable and depends on the type of diet: reported values are 15%–49% [14–16]. The fatty acid composition of the larvae also depends on the fatty acid composition of the diet. To reduce the feeding cost of black soldier flies, animals, and poultry feces or food waste are commonly used as the diet. This leads to the unbalanced fatty acid composition of larvae, especially small amounts of n-3 PUFAs. A study on turbot (*Psetta maxima*) discovered that fish growth decreased as the percentage of BSFLM in test diets increased [17]. In addition, BSFLM impaired the hepatopancreatic structure of *L. vannamei* when the replacement reached 60% [18]. These related studies demonstrated that the replacement level of BSFLMs should be cautiously implemented.

Given its high-market demand and nutritional value, the Chinese mitten crab (*Eriocheir sinensis*) has increasingly become an economically critical freshwater species [19]. In China, the production of *E. sinensis* exceeded 770,000 tons in 2020 [20]. With the continuous expansion of the culture scale, the demand for feed protein sources has increased. Seeking efficient and economical protein sources have become an urgent issue in *E. sinensis* farming. To our knowledge, the effects of BSFLM replacement on growth performance and hepatopancreas health in *E. sinensis* have not been reported. Therefore, this study evaluated the effects of BSFLM, which partially replaced fish meal in the diet, on the growth performance and hepatopancreas health of juvenile *E. sinensis*. The results of this study may contribute to the exploitation and utilization of new protein sources and alleviate the shortage of feed protein sources in the *E. sinensis* farming industry.

## 2. Materials and Methods

### 2.1. Feed Ingredients and Diet Formulation

Six isoproteic (37%) and isolipidic (10%) diets were formulated by replacing 0% (FM), 10% (BSFLM10), 20% (BSFLM20), 30% (BSFLM30), 40% (BSFLM40), and 50% (BSFLM50) of fish meal with BSFLM. The protein sources were fish meal, soybean, cottonseed, and BSFLM (crude protein, 42%; crude lipid, 27%) (Leijian Technology Company, Sichuan, China). Fish oil, soybean oil, soybean lecithin, and cholesterol were the main lipid sources. Raw materials were ground and sieved through a 40-*μ*m mesh. All dry ingredients were ground and blended thoroughly before oil was added. Deionized water (300 mL/kg diet) was added to the mixture, which was then wet-extruded into 2.5-mm-diameter pellets using a double helix plodder (F-26, SCUT industrial factory, Guangdong, China). The pellets were spread out and dried by blowing air at room temperature until reaching approximately 10% moisture and then stored in sealed polyethylene bags at −20°C. The ingredients and proximate compositions of the six experimental diets are shown in Table 1.

### 2.2. Experimental Crabs and Procedure

A 56-day feeding trial was conducted at the Zhejiang Institute of Freshwater Fisheries (Huzhou, Zhejiang, China). The juvenile *E. sinensis* were purchased from a local crab farm in Chongming (Shanghai, China). Before the feeding trial, crabs were temporarily reared in polyethylene tanks (2,000 L) fed commercial feed for 1 week to acclimate to indoor culture conditions. Subsequently, 960 crabs (0.70 ± 0.01 g, mean weight ± SEM) with intact limbs were randomly allocated to 24 white polyethylene tanks of 300-L each (four tanks per treatment and 40 crabs for each tank). Each tank was supplied with four arched tiles and five corrugated plastic pipes (2.5 cm in diameter and 8.0-cm long) as hide-outs to reduce attacks among crabs. The freshwater used in this study went through a filtration system (Xinyi Water Treatment Equipment Factory, Huzhou, China) and was completely aerated. The crabs were fed two times daily (15:00 and 21:00) at a daily ratio of 4% body weight, and food consumption was recorded during a 56-day feeding trial. Feed residue and excrement were cleaned by siphon, and approximately 50% of the water in each tank was exchanged once a day. The water temperature was kept within 25–27°C, pH within 7.6–8.4, ammonia nitrogen at <0.05 mg/L, and dissolved oxygen at >7.0 mg/L throughout the feeding trial.

### 2.3. Sample Collection

At the end of the trial, all crabs were fasted for 24 hr, counted and weighed to calculate the survival rate (SR), weight gain rate (WGR), and specific growth rate (SGR). Then, four crabs at the intermolt period were randomly collected from each tank and kept at −20°C for the analysis of whole-body proximate composition. The hepatopancreases from four crabs were collected and kept at −20°C for analysis of proximate composition and fatty acid composition. The hepatopancreas from eight crabs was collected, immediately frozen in a nitrogen canister, and finally stored at −80°C to determine the enzyme activities and RNA isolation. Pretreatment of the hepatopancreas for enzyme activity detection was the same as described by Lin et al. [21]. WGR, SGR, and SR were evaluated using the following formulae:

(1) $$\begin{array}{c} \text{WGR, (\%)}=\text{[Final weight − initial weight]}/\text{Initial weight}\times 100, \end{array}$$

(2) $$\begin{array}{c} \text{SGR, (\%) }{\text{day}}^{-1}=\text{[ln(final weight) − ln(initial weight)]}\times 100/\text{days}, \end{array}$$

(3) $$\begin{array}{c} \text{SR, (\%)}=\text{(Final crab number/initial crab number)}\times 100. \end{array}$$

### 2.4. Diet, Whole-Body, and Hepatopancreas Composition Analysis

The proximate composition of the diets and the whole-body and hepatopancreas of the crabs were analyzed following the same methods described by Bu et al. [22]. The diets and crabs were dried to a constant weight at 105°C to analyze the moisture. Dried samples were ground for subsequent assays. The crude protein content of the diets, whole-body, and hepatopancreas was measured by the Kjeldahl method (8200, Kjeltec, Foss, Sweden). The crude lipids of the diets, whole-body, and hepatopancreas were extracted with a chloroform/methanol mixture and a 0.37 mol/L KCl solution following the method of Bligh and Dyer [23] and dried in a vacuum drying oven (DZF-6050, Jinghong, Ltd., Shanghai, China) before gravimetry [24]. The ash content of the diets and whole-body was analyzed by carbonizing completely on a heating plate (TR-30A, SuDa, China) and incinerating in a muffle furnace (PCD-E3000 Serials, Peaks, Japan) at 550°C for 6 hr.

The amino acid compositions of the diets (Table 2) were measured by a commercial laboratory (Willtest, Sichuan, China). Diet lipids and hepatopancreas lipids for fatty acid determination were extracted from the untreated samples following the same method as crude lipid extraction. The fatty acid methyl esters were obtained from diet lipids and hepatopancreas lipids by saponification with methanolic KOH (0.5 mol/L) and derivatization with 14% boron trifluoride-methanol. The fatty acid methyl esters (1.0 *μ*L) were dissolved in *n*-hexane (GC residue analysis, CNW, China) and analyzed by gas chromatography–mass spectrometry analysis (GCMS-QP2010 SE, Shimadzu Co., Kyoto, Japan) with a gas chromatographic column (SH-Rt-2560, 0.25 mm ID, 0.20 *μ*m df, 100 m, Shimadzu, USA). The speed of the helium carrier gas was 1.7 mL/min. The injector and flame ionization detector temperatures were 250°C and 200°C, respectively. The program was 120°C for 1 min and 240°C for 30 min, and the total measurement time was 61 min for each sample. The identified fatty acids were expressed as the area percentage of total fatty acids. The fatty acid profiles of diet lipids and hepatopancreas lipids in each group are shown in Tables 3 and 4.

### 2.5. Biochemical Analysis

The hepatopancreas samples were weighed and homogenized in 10 volumes (*v*/*w*) of ice-cooled 0.85% saline solution. The homogenate was centrifuged at 1,500 rpm (5415 R, Eppendorf, Germany) at 4°C for 30 min, and the upper lipid layer was discarded. The supernatant was carefully collected and stored at −80°C until detection. All indicators were measured by the diagnostic reagent kits (Nanjing Jiancheng Bioengineering, Nanjing, China): malondialdehyde (MDA, Cat. No. A003–1) content, total antioxidant capacity (T-AOC, Cat. No. A015–2-1), total superoxide dismutase (SOD, Cat. No. A001–3), and glutathione peroxidase (GSH-Px, Cat. No. A005–1).

### 2.6. Histological Analysis of the Hepatopancreas

Hepatopancreas tissue samples were fixed in 4% paraformaldehyde solution. The samples were dehydrated, washed, and equilibrated using ethanol, toluene, and xylene. Then, the samples were embedded in paraffin and cut at a thickness of 5 *μ*m using a rotary microtome. The hepatopancreas slices were stained with hematoxylin and eosin (HE) and examined on a microscope (BX51, Olympus, Japan).

### 2.7. Analysis of Gene Expression in the Hepatopancreas

Total RNA from the hepatopancreas was isolated by using RNAiso Plus (Takara, Dalian, China). The purity and concentration of total RNA were estimated by the spectrophotometry at A260 and 280 nm using a NanoDrop 2000 spectrophotometer (Thermo, Wilmington, USA). First-strand cDNA was synthesized using a FastKing RT Kit (with gDNase; Tiangen, Beijing, China) according to the manufacturer's instructions. The specific primers for genes are given in Table 5. Real-time quantitative PCR was carried out in a volume of 20 *μ*L, including 10 *μ*L of 2xSYBR qPCR Mix, 0.4 *μ*L of 10 *μ*M forward and reverse primers, and 2 *μ*L of diluted cDNA and 7.2 *μ*L of DEPC-water with a SYBR Green RT‒PCR kit (PC3302, Aidlab, Beijing, China) using a CFX96 real-time PCR system (Bio-Rad, Richmond, CA). The PCR steps included 94°C for 3 min, then at 94°C for 10 s, 60°C for 30 s for 40 cycles, and a melting curve step from 60 to 95°C at an incremental rate of 0.5°C/s. Five dilutions of the cDNA samples (in triplicate) were used to build the standard curve. The amplification efficiency was determined using *E*=10^(−1/Slope)^–1 [25]. The amplification efficiencies of the target genes ranged from 95% to 105%. The housekeeping gene *β-actin* and ubiquitin/ribosomal *S27* fusion protein (*S27*) were employed as reference genes, and the stability of *β-actin* and *S27* expression was confirmed [26]. In this study, the FM group was used as the reference, and the expression of each target gene in the other treatment was expressed as the fold change relative to the control group. The relative expression levels of target genes were analyzed by the 2^−*ΔΔ*Ct^ algorithm [27].

### 2.8. Statistical Analysis

Statistical analysis was implemented using SPSS 20.0 software (Chicago, IL, USA). Homogeneity of variance was checked before a one-way ANOVA, followed by Duncan's multiple comparison tests to assess the significant differences among the means (significance level *P* < 0.05). Data are presented as the means and the pooled standard error of the means (SEM).

## 3. Results

### 3.1. Growth Performance

The effects of different levels of dietary BSFLM on SR, WGR, and SGR are given in Table 6. WGR and SGR were linearly and quadratically affected by the different levels of dietary BSFLM (*P* < 0.05). There were no differences in the SR among all groups (*P* > 0.05). Compared with FM, BSFLM10, BSFLM20, BSFLM30, and BSFLM40 did not significantly affect WGR and SGR (*P* > 0.05). The WGR and SGR of the crabs fed BSFLM50 were lower than those fed FM (*P* < 0.05).

### 3.2. Whole-Body and Hepatopancreas Proximate Composition

The whole-body proximate compositions of the crabs fed different levels of BSFLM are presented in Table 7. The whole-body and hepatopancreas proximate compositions were not linearly or quadratically affected by the different levels of dietary BSFLM (*P* > 0.05). Replacing fish meal with BSFLM did not significantly affect the whole-body and hepatopancreas proximate composition (*P* > 0.05).

### 3.3. Fatty Acid Composition of the Hepatopancreas

The fatty acid composition of the hepatopancreas in the crabs fed different levels of BSFLM is given in Table 4. The current study showed that the levels of saturated fatty acids (SFA), monounsaturated fatty acids (MUFA), polyunsaturated fatty acids (PUFA), and highly unsaturated fatty acid (HUFA) were linearly and quadratically affected by dietary BSFLM (*P* < 0.05). The crabs fed BSFLM had higher SFA levels than those fed FM (*P* < 0.05). The levels of C12 : 0 in the hepatopancreas increased with increasing dietary BSFLM, while the levels of C15 : 0, C16 : 0, C17 : 0, and C18 : 0 in the hepatopancreas were negatively correlated with the increasing dietary BSFLM (*P* < 0.05). The crabs fed BSFLM20, BSFLM30, BSFLM40, and BSFLM50 had higher MUFA levels in the hepatopancreas than those fed FM (*P* < 0.05). The C18 : 1n-9 level was higher in BSFLM50 than in the other groups (*P* < 0.05), and crabs fed BSFLM30, BSFLM40, and BSFLM50 had a higher level than those fed FM, BSFLM10 and BSFLM20 (*P* < 0.05). Compared with the crabs fed FM, those fed BSFLM20, BSFLM30, BSFLM40, and BSFLM50 had lower PUFA levels in the hepatopancreas (*P* < 0.05). Additionally, the C18 : 2n-6 level decreased in the crabs fed BSFLM40 and BSFLM50 compared with those fed FM (*P* < 0.05). The levels of HUFAs, C20 : 4n-6 and C22 : 5n-3 in crabs fed BSFLM were lower than those in the crabs fed FM (*P* < 0.05). The levels of C18 : 3n-3, C20 : 5n-3, and C22 : 6n-3 in the hepatopancreas were decreased in the crabs fed BSFLM20, BSFLM30, BSFLM40, and BSFLM50 compared with those fed FM (*P* < 0.05). The value of n-3 fatty acids/n-6 fatty acids was decreased in the crabs fed BSFLM20, BSFLM30, BSFLM40, and BSFLM50 when compared with those fed FM (*P* < 0.05).

### 3.4. Hepatopancreas Antioxidant Indices

The effects of different levels of dietary BSFLM on the antioxidant indices in the hepatopancreas are shown in Table 8. The present study indicated that the MDA content, SOD activity, GSH-Px activity, and T-AOC activity in the hepatopancreas were linearly and quadratically affected by the different dietary BSFLM levels (*P* < 0.05). Compared with FM, BSFLM30, BSFLM40, and BSFLM50 decreased the SOD and GSH-Px activities in the hepatopancreas (*P* < 0.05). The crabs fed BSFLM50 had lower T-AOC activity in the hepatopancreas than those fed FM (*P* < 0.05). Additionally, compared with FM, BSFLM50 decreased the MDA content in the hepatopancreas (*P* < 0.05).

### 3.5. Gene Expression Related to Nonspecific Immunity

**Figure 1:**
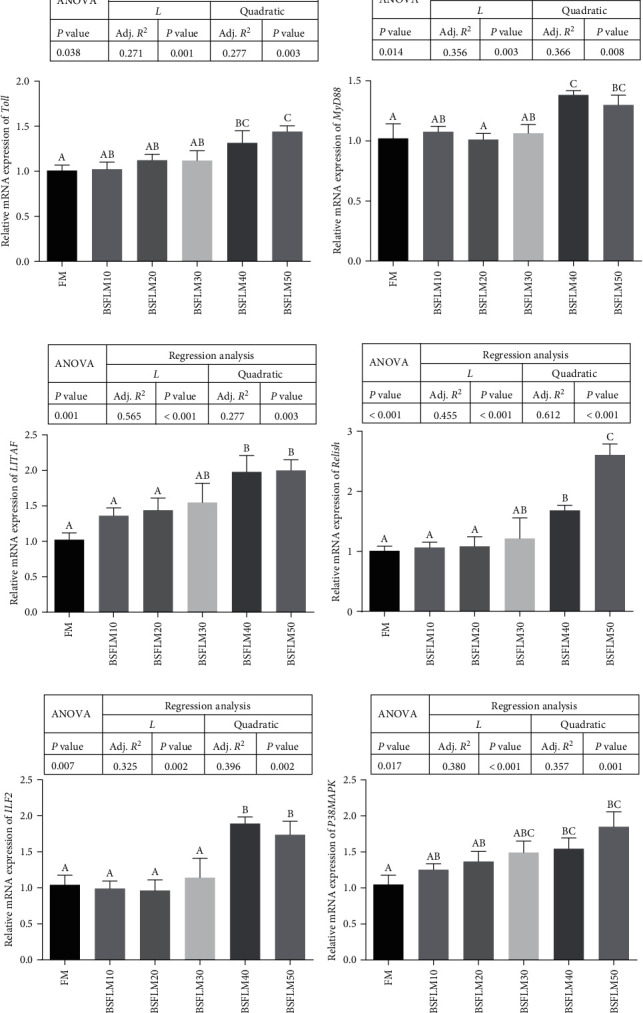
Relative mRNA expression levels of immunity-related genes in the hepatopancreas of juvenile *E*. *sinensis* fed different experimental diets. (a) Toll. (b) *MyD88*, myeloid differentiation factor 88. (c) *LITAF*, lipopolysaccharide-induced TNF factor. (d) *Relish*. (e) *ILF2*, interleukin enhancer binding factor 2. (f) *p38MAPK*, p38 mitogen-activated protein kinase. Adj. *R*^2^, adjusted *R* square; *L*, linear trend; Quadratic, quadratic trend. Different letters show significant differences among treatments (*P* < 0.05).

The expression of genes related to nonspecific immunity (*Toll, MyD88, LITAF, Relish, ILF2*, and *p38MAPK*) in the hepatopancreas is presented in Figure 1. The *Toll, MyD88, LITAF, Relish, ILF2*, and *p38MAPK* genes were linearly and quadratically affected by dietary BSFLM (*P* < 0.05). Compared to the crabs fed FM, the crabs fed BSFLM40 and BSFLM50 had higher gene expression of *Toll, MyD88, LITAF, Relish, ILF2*, and *p38MAPK* in the hepatopancreas (*P* < 0.05).

### 3.6. Histological Analysis of the Hepatopancreas

**Figure 2:**
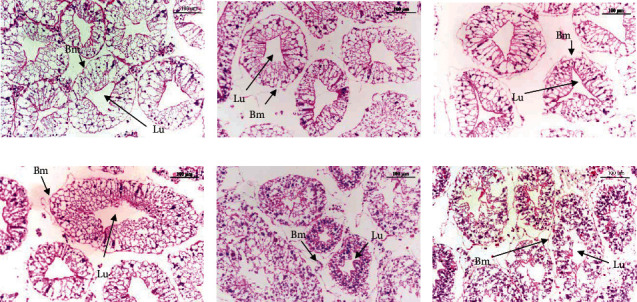
Histopathological analysis of the hepatopancreas of juvenile *E. sinensis* fed different experimental diets. (a) FM, (b) BSFLM10, (c) BSFLM20, (d) BSFLM30, (e) BSFLM40, and (f) BSFLM50. Scale = 100 *μ*m. Lu, lumen; Bm, basal membrane.

The crabs fed FM had normal hepatopancreatic structures with clear lumens, columnar hepatopancreas cells, circular cell nuclei at the base, and a basal membrane with structural integrity (Figure 2(a)). As the inclusion of BSFLM increased, deformation of the hepatopancreatic structure, widened lumens, and loss of integrity of the basal membrane were observed (Figure 2(b)–2(e)). The hepatopancreas of the crabs fed BSFLM50 was severely damaged, and the cell contents were scattered in the cells (Figure 2(f)).

## 4. Discussion

In this study, the replacement of fish meal by BSFLM did not affect the amino acid composition of the diet, as presented in Table 2. This is consistent with the report that BSFLM has a similar amino acid pattern to fish meal [15]. In that case, the effects on crabs were less likely related to the amino acid composition of the protein resource. Compared to fish meal, BSFLM contained almost no n-3 PUFAs. It was rich in SFAs (C12 : 0 and C16 : 0), MUFAs (C18 : 1n-9), and PUFAs (18 : 2n-6). Moreover, due to the high lipid content (27%, DM) of BSFLM, dietary fish oil was also replaced with black soldier fly larvae oil. In BSFLM50, the addition of fish oil was down to 0%. Hence, the fatty acid composition of the diet was significantly changed by the addition of BSFLM, as seen in the fatty acid composition data of Table 3. Increasing levels of BSFLM led to a significant increase in dietary SFAs, such as C12 : 0, and a decrease in n-3 PUFAs, such as C18 : 3n-3, C20 : 5n-3, C22 : 5n-3, and C22 : 6n-3. n-3 PUFAs can improve the absorption, digestion, and transport of nutrients, enhancing the molting and development of swimming crabs (*Portunus trituberculatus*) [28], and they are important for supporting growth performance [29, 30]. In this study, when the replacement of fish meal was not higher than 40%, there was no impairment in the growth performance of crabs. However, the growth performance significantly declined when the replacement reached 50%. The diminished performance in BSFLM50 is more likely due to increased n-3 PUFA deficiencies. A study demonstrated that replacing 30% of fish oil with black soldier fly larvae oil did not affect growth performance while replacing 60% significantly decreased growth performance in juvenile *Totoaba macdonaldi* [31].

In our study, the value of whole-body and hepatopancreas lipid content reached the highest in BSFLM20 and decreased afterward. This may be the result of a combination of factors. Saturated fatty acids are not easily utilized and tend to accumulate in the hepatopancreas [32]. On the other hand, chitin and its derivatives can bind with triacylglycerol and cholesterol and play a critical role in decreasing fatty acid synthesis and increasing the hydrolysis of lipoproteins and triglycerides in the liver [33]. A high dose of chitin and its derivatives can also reduce lipid absorption [34]. As the dietary chitin content increased, lipid absorption and synthesis were inhibited. These findings might explain the variation in lipid content in the whole-body and hepatopancreas. Overall, the absorption and utilization of BSFLM by juvenile *E. sinensis* warrants further study.

Crustaceans have a set of antioxidant enzymes, such as GSH-Px and SOD, to offset ROS toxicity [35]. MDA is the product of lipid peroxidation and directly reflects the level of lipid peroxidation [36]. The MDA content in the hepatopancreas decreased significantly compared with that of crabs fed FM when the replacement level reached 50%. PUFAs are allylic or bis-allylic, which makes them readily susceptible to autoxidation [37]. Therefore, PUFAs in the diet are easily oxidized and produce reactive oxygen species (ROS). A study on rats demonstrated a significant increase in lipid peroxidation products when fed a fish oil-containing diet for 4 weeks [38]. Our results showed that the relative content of PUFAs in the hepatopancreas lipid dramatically decreased as dietary BSFLM increased. The lower PUFA content in the hepatopancreas would decrease the risk of lipid peroxidation. The results showed that the activities of antioxidant enzymes (SOD and GSH-Px) in the hepatopancreas decreased as the dietary BSFLM level reached 30%, and the activity of T-AOC in the hepatopancreas was significantly lower than that in the hepatopancreas of crabs fed FM when replacement reached 50%. This might be because replacing fish meal with BSFLM decreased dietary PUFA levels and consequently decreased ROS production. The activities of antioxidant enzymes were not activated. In contrast, a study in European seabass (*Dicentrarchus labrax*) demonstrated a significant elevation in SOD activities and MDA content in serum when the replacement was over 25% [39]. Another study reported no significant difference in MDA levels and the activities of SOD and GSH-Px in hepatic and renal tissues of rainbow trout (*Oncorhynchus mykiss*) when dietary fish meal was partly replaced by BSFLM [40]. The difference in antioxidant responses of aquatic animals toward the dietary inclusion of BSFLM may be attributed to several factors, such as different species, different feeding periods, inclusion doses of BSFLM, the experimental setup, or the feeding regimes. Thus, further research needs to be performed to better understand the potential antioxidant mechanisms of BSFLM in the aquatic animals.

Chitin in BSFLM is a potent stimulator of innate immune responses. This activation is mediated mainly via the toll-like receptor-2–nuclear factor-*κ*B (*TLR-2-NF-κB*) pathway [41]. In this study, we measured the expression of innate immune-related genes in the hepatopancreas, and the results showed that the expression of *Toll* and *MyD88* was upregulated in BSFLM40 and BSFLM50, demonstrating that dietary BSFLM could activate nonspecific immunity in crabs by activating the *Toll* pathway in the hepatopancreas. As an essential transcription factor, *NF-κB* can trigger the release of proinflammatory cytokines to induce inflammation [42]. *Relish* is the homolog of *NF-κB* in *E. sinensis* and has the same function as *NF-κB* [43]. Moreover, the activation of *p38MAPK* can also release proinflammatory cytokines [44]. Thus, dietary BSFLM might promote the expression of *Relish* and *p38MAPK* to release proinflammatory cytokines in the hepatopancreas. *LITAF* is a critical transcription factor that binds to promoter regions and promotes the expression of proinflammatory cytokines such as *TNF-α* and *IL-2* [45, 46]. *TNF-α* is an important mediator of chronic inflammation as a pleiotropic cytokine [47]. Research has found that soybean antigen induces the expression of *LITAF* to impair the health of Chinese mitten crab [48]. In the present study, crabs fed high levels of BSFLM also had higher expression of *LITAF* and *ILF2*, indicating that BSFLM might promote the expression of proinflammatory cytokines. In addition, n-3 PUFAs are considered important components of the cell membrane, and they can effectively maintain cell membrane homeostasis and perform anti-inflammatory functions [49]. The n-6 PUFAs give rise to the eicosanoid family of mediators (prostaglandins, thromboxanes, leukotrienes, and related metabolites). These have inflammatory actions in their own right and regulate the production of other mediators, including proinflammatory cytokines. A high ratio of dietary n-3/n-6 PUFAs could improve the inflammation [50]. The decrease in n-3 PUFAs and a low ratio of n-3/n-6 PUFAs in diets might aggravate the activation of the inflammatory response in the hepatopancreas. The hepatopancreatic histopathological structural change is an indispensable indicator of the health status of invertebrates [51, 52]. In our study, pathological injury was distinctly observed in the hepatopancreas when the replacement of fish meal with BSFLM reached 50%. This was similar to the observations in *L. vannamei* [18] and Jian carp [53]. The variation in n-3 PUFAs may partly explain the observation among all groups [53]. Additionally, excessive amounts of proinflammtatory cytokines result in hepatopancreas injury. Overall, high substitution of fish meal by BSFLM should be avoided in *E. sinensis* in case of damage to the hepatopancreas.

The replacement of 40% fish meal with BSFLM did not significantly impact the growth performance of crabs. However, it was observed that antioxidant enzyme activities and immunity were significantly affected when the replacement level reached 40% or even 30%. This could be attributed to the rapid change in biochemical indexes compared to growth performance. It is important to note that sometimes growth may not be significantly affected, but the health status can already be at risk [53, 54]. Therefore, besides focusing on the growth, attention should also be given to maintain the health of juvenile *E. sinensis* by considering fish meal replacement with BSFLM.

## 5. Conclusions

This study demonstrates that a 40% replacement level of dietary fish meal by BSFLM did not impact the growth performance of juvenile *E. sinensis*. However, BSFLM activated the immune responses when the replacement level reached 40%. Moreover, 50% replacement resulted in structural impairment to the hepatopancreas. Based on the growth performance and hepatopancreas health, it is recommended that the replacement of fish meal by BSFLM be not more than 30% in the juvenile *E. sinensis* diet.

## Figures and Tables

**Table 1:** Ingredient formulation of the feed formula (g/kg dry basis) and diet proximate composition (% dry matter, *n* = 3).

Ingredients	Experimental diets					
	FM	BSFLM10	BSFLM20	BSFLM30	BSFLM40	BSFLM50
Fish meal^1^	350	315	280	245	210	175
Black soldier fly larvae meal^2^	0	57	113	169	226	282
Soybean meal	130	130	130	130	130	130
Cotton meal	130	130	130	130	130	130
Fish oil	30	24	18	12	6	0
Soybean oil	30	24	18	12	6	0
Lecithin	5	5	5	5	5	5
Cholesterol	5	5	5	5	5	5
*α*-Starch	170	170	170	170	170	170
Cellulose	54	44	35	26	16	7
Choline chloride	5	5	5	5	5	5
Butylated hydroxytoluene	1	1	1	1	1	1
Vitamin premix^3^	40	40	40	40	40	40
Mineral premix^4^	20	20	20	20	20	20
Alginate-Na	30	30	30	30	30	30
Total	1,000	1,000	1,000	1,000	1,000	1,000
Proximate composition (%)						
Moisture	9.08	9.60	10.26	9.80	9.92	10.29
Crude protein	38.02	37.76	38.25	37.94	37.52	38.25
Crude lipid	9.54	10.00	9.57	9.56	9.48	9.58
Crude ash	11.19	11.63	11.67	11.51	11.72	11.66

^1^The crude protein concent of fish meal is 68.52% (dry matter). ^2^The crude protein content of black soldier fly larvae meal is 42.61% (dry matter). ^3^Vitamin mix (per 100 g premix) retinol acetate, 0.043 g; thiamin hydrochloride, 0.15 g; riboflavin, 0.0625 g; niacin, 0.3 g; Ca pantothenate, 0.3 g; pyridoxine hydrochloride, 0.225 g; ascorbic acid, 0.5 g; para-aminobenzoic acid, 0.1 g; folic acid, 0.025 g; biotin, 0.005 g; cholecalciferol, 0.0075 g; *α*-tocopherol acetate, 0.5 g; menadione, 0.05 g; inositol, 1 g. All ingredients were filled with *α*-cellulose to 100 g. ^4^Mineral premix (per 100 g premix): KH_2_PO_4_, 21.5 g; NaH_2_PO_4_, 10.0 g; Ca(H_2_PO_4_)_2_, 26.5 g; CaCO_3_, 10.5 g; KCl, 2.8 g; MgSO_4_·7H_2_O, 10.0 g; AlCl_3_·6H_2_O, 0.024 g; ZnSO_4_·7H_2_O, 0.476 g; MnSO_4_·6H_2_O, 0.143 g; KI, 0.023 g; CuCl_2_··2H_2_O, 0.015 g; CoCl_2_··6H_2_O, 0.14 g; calcium lactate, 16.50 g; Fe-citrate, 1 g. All ingredients were diluted with *α*-cellulose to 100 g.

**Table 2:** Amino acid composition of six experimental diets (% dry matter, *n* = 2).

Amino acids	Experimental diets					
	FM	BSFLM10	BSFLM20	BSFLM30	BSFLM40	BSFLM50
EAA						
Lys	2.53	2.49	2.57	2.54	2.55	2.44
Met	0.84	0.73	0.79	0.75	0.81	0.71
Leu	2.88	2.81	2.88	2.85	2.86	2.81
Ile	1.64	1.62	1.65	1.65	1.65	1.62
Arg	2.80	2.77	2.81	2.75	2.79	2.73
Phe	1.77	1.78	1.79	1.78	1.77	1.75
Thr	1.64	1.60	1.65	1.61	1.62	1.58
Val	1.92	1.99	1.96	1.98	1.94	1.98
His	1.15	1.18	1.18	1.17	1.15	1.16
NEAA						
Asp	3.83	3.85	3.87	3.87	3.82	3.81
Ser	1.63	1.66	1.69	1.64	1.60	1.64
Glu	6.26	6.21	6.29	6.22	6.20	6.08
Gly	2.11	2.04	2.10	2.05	2.09	2.01
Ala	2.26	2.28	2.30	2.28	2.26	2.23
Cys	0.45	0.43	0.43	0.45	0.43	0.42
Pro	1.73	1.86	1.82	1.84	1.76	1.89
Tyr	1.09	1.34	1.21	1.26	1.16	1.38
TAA	36.53	36.64	36.99	36.69	36.46	36.24
* Σ*EAA	14.64	14.48	14.71	14.54	14.59	14.34
* Σ*NEAA	19.36	19.67	19.71	19.61	19.32	19.46
* Σ*EAA/*Σ*NEAA	0.76	0.74	0.75	0.74	0.76	0.74
* Σ*EAA/*Σ*TAA	0.40	0.40	0.40	0.40	0.40	0.40

EAA, essential amino acids; NEAA, nonessential amino acids; TAA, total amino acids.

**Table 3:** Fatty acid composition of ingredients and six experimental diets (% total fatty acids, *n* = 3).

Fatty acid	BSFLM	Fish meal	FM	BSFLM10	BSFLM20	BSFLM30	BSFLM40	BSFLM50
C12 : 0	17.39	ND	ND	10.34	16.74	24.20	29.51	33.45
C14 : 0	5.12	6.91	8.33	8.08	7.68	7.62	7.38	7.26
C15 : 0	0.17	0.49	0.68	0.39	0.53	0.40	0.28	0.25
C16 : 0	15.89	20.47	26.63	24.23	22.34	20.72	19.41	18.20
C16 : 1	3.74	8.84	7.73	6.66	5.65	4.81	4.20	3.59
C17 : 0	0.29	0.65	0.52	0.48	0.35	0.34	0.24	0.22
C17 : 1	0.40	ND	0.23	0.19	0.10	0.16	0.17	0.15
C18 : 0	4.82	5.87	4.14	3.93	4.06	3.27	3.28	2.89
C18 : 1n-9	24.19	15.11	16.18	15.67	15.39	15.05	14.78	14.89
C18 : 2n-6	24.12	0.45	24.75	21.50	20.02	18.03	16.52	15.63
C20	ND	0.96	0.51	0.45	0.32	0.22	0.06	0.10
C18 : 3n-3	2.07	2.71	2.95	2.57	2.34	2.03	1.81	1.69
C20 : 4n-6	0.54	1.85	0.43	0.30	0.30	0.29	0.25	0.23
C20 : 5n-3	0.52	17.34	5.02	3.80	3.13	2.19	1.64	1.18
C22 : 5n-3	ND	2.35	0.30	0.21	0.15	0.08	0.05	ND
C22 : 6n-3	0.74	16.00	1.63	1.26	1.02	0.67	0.50	0.31
SFA	43.68	35.35	40.81	47.88	52.00	56.75	60.16	62.36
MUFA	28.33	23.95	24.14	22.51	21.13	20.01	19.15	18.62
PUFA	27.99	40.70	35.07	29.63	26.94	23.28	20.76	19.03
HUFA	3.87	40.25	10.32	8.13	6.93	5.26	4.24	3.40
∑n-3FA	3.33	38.40	9.89	7.83	6.63	4.97	3.99	3.17
∑n-6FA	24.66	2.30	25.18	21.80	20.31	18.32	16.77	15.86
∑n-3FA/∑n-6FA	0.52	18.30	5.53	4.24	3.45	2.41	1.70	1.28

SFA, saturated fatty acid; C12 : 0, C14 : 0, C15 : 0, C16 : 0, C17 : 0, C18 : 0; MUFA, monounsaturated fatty acid; C16 : 1, C17 : 1, C18 : 1; PUFA, polyunsaturated fatty acid; C18 : 2n-6, C18 : 3n-6, C18 : 3n-3; HUFA, highly unsaturated fatty acid; C20 : 3n-6, C20 : 4n-6, C20 : 5n-3, C22 : 6n-3; n-3 FA, n-3 fatty acid: C18 : 3n-3, C20 : 5n-3, C22 : 5n-3, C22 : 6n-3; n-6 FA, n-6 fatty acid: C18 : 2n-6, C18 : 3n-6, C20 : 4n-6; ND, not detected.

**Table 4:** Fatty acid composition of the hepatopancreas (% total fatty acids, *n* = 4) in juvenile *E*. *sinensis* fed different diets.

Fat acids	Experimental diets						ANOVA	Regression analysis			
	FM	BSFLM10	BSFLM20	BSFLM30	BSFLM40	BSFLM50		Linear		Quadratic	
							*P*-value	Adj. *R*^2^	*P*-Value	Adj. *R*^2^	*P*-Value
C12 : 0	ND^g^	2.30 ± 0.00^a^	4.39 ± 0.08^b^	4.67 ± 0.15^b^	8.01 ± 0.35^c^	9.33 ± 0.05^d^	<0.001	0.938	<0.001	0.938	<0.001
C14 : 0	4.59 ± 0.21	5.00 ± 0.49	5.36 ± 0.12	5.16 ± 0.23	5.29 ± 0.24	4.99 ± 0.37	0.662	−0.031	0.531	0.055	0.233
C15 : 0	0.58 ± 0.02^b^	0.53 ± 0.03^b^	0.43 ± 0.02^a^	0.44 ± 0.02^a^	0.41 ± 0.04^a^	0.39 ± 0.01^a^	<0.001	0.606	<0.001	0.646	<0.001
C16 : 0	30.32 ± 0.56^c^	29.02 ± 1.22^bc^	27.60 ± 0.24^ab^	27.84 ± 0.64^ab^	26.63 ± 0.50^a^	26.39 ± 0.80^a^	0.010	0.515	<0.001	0.516	0.001
C16 : 1	10.65 ± 0.20	10.95 ± 0.34	11.22 ± 0.15	10.84 ± 0.49	10.27 ± 0.32	10.33 ± 0.05	0.374	0.048	0.185	0.123	0.136
C17 : 0	0.54 ± 0.05^b^	0.47 ± 0.06^b^	0.26 ± 0.01^a^	0.30 ± 0.03^a^	0.24 ± 0.01^a^	0.30 ± 0.03^a^	<0.001	0.455	<0.001	0.678	<0.001
C17 : 1	0.35 ± 0.01	0.34 ± 0.01	0.35 ± 0.02	0.31 ± 0.02	0.33 ± 0.01	0.32 ± 0.01	0.399	0.085	0.096	0.041	0.253
C18 : 0	3.50 ± 0.15^b^	3.00 ± 0.14^a^	2.62 ± 0.08^a^	2.91 ± 0.26^a^	2.76 ± 0.09^a^	2.60 ± 0.20^a^	0.012	0.299	0.003	0.351	0.004
C18 : 1n-9	26.65 ± 0.30^a^	26.70 ± 0.19^a^	26.92 ± 0.27^a^	29.88 ± 0.69^b^	30.13 ± 1.16^b^	34.55 ± 0.06^c^	<0.001	0.752	<0.001	0.860	<0.001
C18 : 2n-6	15.69 ± 0.40^c^	15.85 ± 0.39^c^	15.04 ± 0.32^bc^	14.84 ± 0.35^abc^	14.46 ± 0.15^ab^	13.84 ± 0.15^a^	0.005	0.550	<0.001	0.540	<0.001
C18 : 3n-3	0.57 ± 0.02^c^	0.54 ± 0.05^c^	0.38 ± 0.00^b^	0.36 ± 0.02^b^	0.29 ± 0.01^a^	0.27 ± 0.01^a^	<0.001	0.830	<0.001	0.846	<0.001
C20 : 0	1.20 ± 0.19	1.28 ± 0.23	1.32 ± 0.09	1.31 ± 0.06	1.29 ± 0.05	1.11 ± 0.12	0.923	−0.062	0.729	−0.029	0.474
C20 : 4n-6	0.69 ± 0.06^b^	0.45 ± 0.01^a^	0.38 ± 0.03^a^	0.49 ± 0.10^a^	0.41 ± 0.07^a^	0.42 ± 0.04^a^	0.026	0.194	0.023	0.291	0.015
C20 : 5n-3	3.57 ± 0.05^e^	2.86 ± 0.07^de^	2.37 ± 0.12^d^	1.94 ± 0.21^c^	1.65 ± 0.08^b^	1.34 ± 0.04^a^	<0.001	0.901	<0.001	0.925	<0.001
C22 : 5n-3	0.16 ± 0.01^b^	0.13 ± 0.01^a^	0.02 ± 0.02^a^	ND^g^	ND^g^	ND^g^	0.001	0.746	0.001	0.833	0.001
C22 : 6n-3	1.55 ± 0.11^d^	1.00 ± 0.07^cd^	0.86 ± 0.07^bc^	0.78 ± 0.17^b^	0.56 ± 0.05^b^	0.43 ± 0.05^a^	<0.001	0.732	<0.001	0.766	<0.001
∑SFA	35.74 ± 0.49^a^	37.75 ± 0.26^b^	39.13 ± 0.34^c^	39.58 ± 0.47^c^	41.89 ± 0.56^d^	42.18 ± 0.06^d^	<0.001	0.876	<0.001	0.878	<0.001
∑MUFA	33.95 ± 0.21^a^	35.48 ± 0.61^ab^	35.81 ± 0.47^b^	36.03 ± 0.52^b^	36.35 ± 0.79^b^	38.32 ± 0.92^c^	0.004	0.531	<0.001	0.508	<0.001
∑PUFA	16.99 ± 0.59^d^	16.36 ± 0.50^cd^	15.42 ± 0.32^bc^	15.20 ± 0.46^abc^	14.73 ± 0.14^ab^	14.12 ± 0.14^a^	0.001	0.689	<0.001	0.680	<0.001
∑HUFA	5.47 ± 0.30^d^	3.91 ± 0.39^c^	3.61 ± 0.23^c^	3.20 ± 0.46^bc^	2.59 ± 0.19^ab^	2.19 ± 0.11^a^	<0.001	0.727	<0.001	0.745	<0.001
∑n-3FA	5.35 ± 0.33^e^	4.27 ± 0.30^d^	3.62 ± 0.20^cd^	3.08 ± 0.39^bc^	2.48 ± 0.13^ab^	2.05 ± 0.08^a^	<0.001	0.841	<0.001	0.851	<0.001
∑n-6FA	16.82 ± 0.48^d^	16.28 ± 0.55^cd^	15.66 ± 0.33^bcd^	15.38 ± 0.39^abc^	14.85 ± 0.18^ab^	14.28 ± 0.12^a^	0.003	0.674	<0.001	0.655	<0.001
∑n-3FA/∑n-6FA	0.32 ± 0.01^e^	0.27 ± 0.01^de^	0.24 ± 0.01^d^	0.18 ± 0.01^c^	0.16 ± 0.00^b^	0.14 ± 0.00^a^	<0.001	0.931	<0.001	0.940	<0.001

The values are the mean ± standard error (*n* = 4). Adj. *R*^2^, adjusted *R*^2^; Linear, linear trend; Quadratic, quadratic trend. Means in the same line with different superscripts are significantly different (*P* < 0.05).

**Table 5:** Primer pair sequences and product sizes of the genes used for real-time PCR.

Genes	Position	Primer sequence	Product size (bp)	References
*β-Actin*	Forward	TCGTGCGAGACATCAAGGAAA	178	Lin et al. [21]
	Reverse	AGGAAGGAAGGCTGGAAGAGTG	—	

*S27*	Forward	CCCCCAAGAAGATCAAGCACA	179	Lin et al. [21]
	Reverse	CAGATGGCAGCGACCACAGTA	—	

*LITAF*	Forward	TAAAGGCAAGGGAGGCTTCG	97	GenBank: KC011816.1
	Reverse	GAATGGAGCTTGAGGTGGCA	—	

*Relish*	Forward	TCAGGATTCGGTGGCAACTC	105	GenBank: GQ871279.1
	Reverse	ATCTGCACTTGGACCGATGG	—	

*ILF2*	Forward	GGGAACTTCGATGCCTGTCA	101	GenBank: GU002546
	Reverse	ATGACCACGATGTCCGCTAC	—	

*Toll*	Forward	CTCCTTCACCTGCCCTAACTGCT	88	GenBank: KC011816.1
	Reverse	CTCCAGTTTGTATTGCTGTGCGAAA	—	

*MyD88*	Forward	GCCATCGCAGTCGCCAAGTT	148	GenBank: KC019316.1
	Reverse	GGCATCCTGTTCATCCAGTTCTGAC	—	

*p38MAPK*	Forward	TGGGAGGTGCCCAAGAGATA	94	GenBank: KF582665.1
	Reverse	TGGTGTTTGTTTTGGCGTCC	—	

*S27*, ubiquitin/ribosomal *S27* fusion protein; *LITAF*, lipopolysaccharide-induced TNF factor; *ILF2*, interleukin enhancer binding factor 2; *MyD88*, myeloid differentiation factor 88; *p38MAPK*, p38 mitogen-activated protein kinase.

**Table 6:** Growth performance of juvenile *E. sinensis* fed different diets.

Experimental diets	Parameter		
	SR (%)	WGR (%)	SGR (%/d)
FM	77.14 ± 1.65	270.14 ± 1.47^bc^	2.34 ± 0.01^bc^
BSFLM10	77.71 ± 4.00	270.88 ± 4.32^bc^	2.34 ± 0.02^bc^
BSFLM20	84.00 ± 3.01	277.19 ± 1.41^c^	2.37 ± 0.01^c^
BSFLM30	82.29 ± 1.71	266.95 ± 2.67^b^	2.32 ± 0.01^b^
BSFLM40	83.71 ± 2.70	266.11 ± 2.19^ab^	2.32 ± 0.01^ab^
BSFLM50	84.34 ± 2.70	259.31 ± 1.76^a^	2.28 ± 0.01^a^
ANOVA			
*P* value	0.759	0.001	0.001
Regression analysis (*n* = 4)			
*L*			
Adj. *R*^2^	0.102	0.220	0.221
* P* value	0.045	0.002	0.002
Quadratic			
Adj. *R*^2^	0.089	0.348	0.349
* P* value	0.102	<0.001	<0.001

The values are the mean ± standard error (*n* = 4). Adj. *R*^2^, adjusted *R*^2^; L, linear trend; Quadratic, quadratic trend. Different letters indicate significant differences (*P* < 0.05).

**Table 7:** Approximate composition (% original substance) of the whole-body and hepatopancreas in juvenile *E. sinensis* fed different diets.

Experimental diets	Parameter					
	Whole-body (%)				Hepatopancreas (%)	
	Moisture	Crude protein	Crude lipid	Crude ash	Crude protein	Crude lipid
FM	66.93 ± 2.31	13.17 ± 0.65	4.06 ± 0.74	0.36 ± 0.01	10.31 ± 0.27	27.32 ± 2.69
BSFLM10	66.17 ± 1.80	13.37 ± 0.70	4.18 ± 0.42	0.37 ± 0.03	9.91 ± 0.28	29.27 ± 3.57
BSFLM20	66.36 ± 0.79	12.92 ± 0.20	4.79 ± 0.08	0.36 ± 0.01	9.47 ± 0.52	34.72 ± 1.38
BSFLM30	66.49 ± 2.13	13.14 ± 0.82	4.01 ± 0.39	0.37 ± 0.01	9.65 ± 0.59	29.26 ± 3.31
BSFLM40	66.65 ± 0.68	13.05 ± 0.29	4.03 ± 0.36	0.36 ± 0.01	9.38 ± 0.16	29.26 ± 1.68
BSFLM50	66.91 ± 1.35	13.33 ± 0.63	4.29 ± 0.25	0.37 ± 0.02	9.47 ± 0.58	26.07 ± 2.79
ANOVA						
*P* value	0.999	0.996	0.743	0.541	0.659	0.322
Regression analysis (*n* = 4)						
*L*						
Adj. *R*^2^	−0.045	−0.053	−0.035	0.015	0.070	−0.018
* P* value	0.906	0.988	0.930	0.278	0.113	0.557
Quadratic						
Adj. *R*^2^	−0.088	−0.105	−0.073	−0.045	0.058	−0.016
* P* value	0.932	0.948	0.984	0.546	0.815	0.500

The values are the mean ± standard error (*n* = 4). Adj. *R*^2^, adjusted *R* square; L, linear trend; Quadratic, quadratic trend. Different letters indicate significant differences (*P* < 0.05).

**Table 8:** Antioxidant parameters in the hepatopancreas of juvenile *E. sinensis* fed different diets.

Experimental diets	Parameter			
	MDA (nmol/mgprot)	SOD (U/mgprot)	GSH-Px (U/mgprot)	T-AOC (nmol/gprot)
FM	7.32 ± 1.04^b^	11.80 ± 0.54^b^	252.98 ± 13.71^b^	38.09 ± 1.75^b^
BSFLM10	5.88 ± 0.32^ab^	10.94 ± 1.05^b^	251.67 ± 16.16^b^	37.96 ± 1.31^b^
BSFLM20	5.85 ± 1.16^ab^	9.70 ± 0.71^ab^	220.05 ± 10.63^ab^	27.95 ± 6.08^ab^
BSFLM30	5.51 ± 0.72^ab^	7.81 ± 0.49^a^	207.16 ± 4.82^a^	27.45 ± 8.70^ab^
BSFLM40	5.39 ± 0.21^ab^	7.68 ± 0.17^a^	206.10 ± 7.14^a^	24.56 ± 2.02^ab^
BSFLM50	4.27 ± 0.18^a^	7.54 ± 1.24^a^	206.66 ± 20.65^a^	21.31 ± 2.68^a^
ANOVA				
*P* value	0.063	0.004	0.015	0.091
Regression analysis (*n* = 4)				
*L*				
Adj. *R*^2^	0.401	0.624	0.242	0.296
* P* value	0.001	<0.001	0.001	0.002
Quadratic				
Adj. *R*^2^	0.369	0.635	0.244	0.269
* P* value	0.006	<0.001	0.002	0.010

The values are the mean ± standard error (*n* = 4). Adj. *R*^2^, adjusted *R*^2^; *L*, linear trend; Quadratic, quadratic trend. Different letters indicate significant differences (*P* < 0.05). MDA, malondialdehyde; SOD, superoxide dismutase; GSH-Px, glutathione peroxidase; T-AOC, total antioxidation capability.

## Data Availability

All data are available upon request by contact with the corresponding author.
